# Serum Soluble B Cell-Activating Factor Is a Non-Invasive Biomarker of Antibody-Mediated Rejection in Kidney Allograft With Satisfactory Risk Stratification Performance But Negligible Diagnostic Value

**DOI:** 10.3389/fimmu.2022.869444

**Published:** 2022-04-13

**Authors:** Shenghui Wu, Xiaojun Su, Qianyu Ye, Yongcheng Wei, Yifang Gao, Mingchuan Huang, Yanxu Chen, Jiali Wang, Qiang Zhang, Qian Fu, Jun Li, Chenglin Wu, Huiting Huang, Bowen Xu, Huanxi Zhang, Longshan Liu, Changxi Wang

**Affiliations:** ^1^ Organ Transplant Center, The First Affiliated Hospital, Sun Yat-sen University, Guangzhou, China; ^2^ Guangdong Provincial Key Laboratory on Organ Donation and Transplant Immunology, Guangzhou, China; ^3^ Department of Nephrology, The First Affiliated Hospital, Sun Yat-sen University, Guangzhou, China; ^4^ Guangdong Provincial International Cooperation Base of Science and Technology (Organ Transplantation), The First Affiliated Hospital, Sun Yat-sen University, Guangzhou, China

**Keywords:** B cell-activating factor (BAFF), antibody-mediated allograft rejection, diagnostic, predictive, kidney transplantation

## Abstract

**Objectives:**

B cell-activating factor (BAFF), which is critical in the activation and differentiation of B cells, is a candidate diagnostic and predictive biomarker for antibody-mediated rejection (ABMR). We aimed to investigate the value of serum soluble BAFF (sBAFF) for the diagnosis and risk stratification of ABMR after kidney transplantation.

**Methods:**

In the diagnostic study, sBAFF level among ABMR (n = 25), T cell-mediated rejection (TCMR) (n = 14), 4 other pathological lesions (n = 21), and stable allograft function group (n = 15) were compared. In the nested case-control study, kidney allograft recipients with *de novo* donor-specific antibody (DSA) or ABMR (n = 16) vs. stable allograft function (n = 7) were enrolled, and sBAFF was measured preoperatively, at D7, M1, M3, M6, M9, M12, M18 posttransplant and at allograft biopsy.

**Results:**

There was no significant difference in sBAFF level at biopsy between ABMR and non-ABMR groups. Longitudinal study showed that the sBAFF levels decreased dramatically at D7 in both groups. The sBAFF level in the DSA group started to increase within M1, while in the stable group, it maintained a low level until M3 and M6. The sBAFF levels of the DSA group were significantly higher than that of the stable group at M1 [1,013.23 (633.97, 1,277.38) pg/ml vs. 462.69 (438.77, 586.48) pg/ml, *P* = 0.005], M3 [1,472.07 (912.79, 1,922.08) pg/ml vs. 561.63 (489.77, 630.00) pg/ml, *P* = 0.002], and M6 [1,217.95 (965.25, 1,321.43) pg/ml vs. 726.93 (604.77, 924.60) pg/ml, *P* = 0.027]. sBAFF levels at M3 had the best predictive value for the DSA/ABMR with the area under the receiver operating characteristic (AUROC) curve value of 0.908. The predictive performance of the maximum (max) change rate from D7 to the peak within M3 was also excellent (AUROC 0.949, *P* = 0.580).

**Conclusion:**

We clarified by a diagnostic study that sBAFF is not a diagnostic biomarker for ABMR in kidney transplantation and revealed by a nested case-control study that sBAFF values at M3 posttransplant and dynamic changes in sBAFF within M3 posttransplant have a good predictive value for the DSA/ABMR. It provides a useful tool for early screening of low-risk patients with negative preoperative DSA for the risk of developing postoperative DSA in kidney allograft recipients.

## Introduction

Antibody-mediated rejection (ABMR) is one of the most serious complications after kidney transplantation, recognized as the main cause of late renal allograft loss (1, 2). Donor-specific antibody (DSA) is the most widely used non-invasive biomarker for ABMR, playing an important role in risk stratification and diagnosis of ABMR (3). On the one hand, preoperative DSA of recipients is one of the few ways to estimate the risk of postoperative ABMR. However, the majority of recipients were negative in preoperative DSA, characterized as low immunological risk. Robust predictive markers, targeted at low-risk recipients, for ABMR are still lacking. On the other hand, DSA has also been regarded as a diagnostic marker for ABMR, but only 30%–40% DSA-positive recipients have been confirmed as ABMR (4). Furthermore, approximately 60% of recipients suffering from ABMR are DSA negative (5). Besides, high economic costs limit the continuous monitoring and adequate use of DSA in the clinics. Therefore, finding a non-invasive biological marker for ABMR remains a priority in the field of transplantation.

There is a lot of evidence indicating that B cells are involved in the development of ABMR (6, 7). B cell-activating factor (BAFF, also known as BLys), a member of the tumor necrosis factor (TNF) family, is an essential regulator of primary B-cell homeostatic modulation. Soluble BAFF (sBAFF) is mainly secreted by innate immune cells, such as monocytes, macrophages, dendritic cells, and neutrophils (8), and performs its function by binding to three distinct receptors, including BAFF receptor (BAFFR), transmembrane activator and calcium modulator and cyclophilin ligand interactor (TACI), and B-cell maturation antigen (BCMA). These receptors are expressed on the surface of B cells at different stages of development (9, 10).

Many studies have investigated the predictive effect of sBAFF on ABMR after kidney transplantation, but the results reported are conflicting (11–13). We performed a meta-analysis that concluded that the kidney allograft recipients with elevated serum sBAFF levels pretransplant and/or posttransplant have greater risk of ABMR (14). However, the vast majority of predictive studies based on a single time point only, for example, preoperative or postoperative point, lack continuous monitoring. Because of low testing cost, BAFF is ideal for continuous detection, while few studies had examined the predictive power of continuously monitoring BAFF for ABMR (15, 16). The time points in these studies were sparse, especially missing the very early postoperative point. In addition, the subjects of previous longitudinal studies were children, whose immune characteristics were different from those of adults. It is necessary to continuously monitor sBAFF levels and explore the value of risk stratification by sBAFF in adults. Not only that, we hypothesized that sBAFF could be valuable in the clinical diagnosis of ABMR after kidney transplantation. However, the diagnostic value of serum sBAFF levels to ABMR has not been extensively investigated. Therefore, we conducted a diagnostic study to evaluate the performance of sBAFF in ABMR diagnosis after kidney transplantation and used the periodical specimen in the specimen bank to explore the dynamic changes of sBAFF levels before the ABMR or DSA.

## Materials and Methods

### Study Design and Patients

This was a single-center study based on a specimen bank and a kidney recipient database. To explore the predictive and diagnostic value of sBAFF levels for ABMR, this study consisted of two parts: the first was a diagnostic study and the second was a nested case-control study.

1) In the first part, participants were selected from the kidney allograft recipients with allograft biopsy and serum samples preserved at the same time in the First Affiliated Hospital of Sun Yat-sen University, Guangzhou, China, from August 2016 to January 2020. All enrolled participants were classified into 6 groups: antibody-mediated rejection (ABMR), T cell-mediated rejection (TCMR), BK polyomavirus nephropathy (BKVN), focal segmental glomerulosclerosis (FSGS), interstitial fibrosis and tubular atrophy (IFTA), and IgA nephropathy (IgAN). The biopsy was evaluated according to the 2015 Banff Kidney Rejection Classification, in which DSA was required to be positive in ABMR and mean fluorescence intensity (MFI) ≥1,000 was considered positive (1). The stable allograft function group was randomly selected from the patients 1) whose urine protein was negative, 2) whose serum creatinine (Scr) was less than 115 umol/L and undulated within ±20% of the mean Scr during the previous year or the period between recovery and enrollment, and 3) with posttransplant serum sample preserved in the specimen bank. They were matched with ABMR recipients in both gender and postoperative time. Besides BK virus infection, patients with other ongoing infections (e.g., cytomegalovirus (CMV), hepatitis B virus (HBV), or hepatitis C virus (HIV) or infections during the past 3 months before enrollment were excluded. Mixed rejection was also excluded. The sample size of each group was determined by calculation (see *Sample Size* section below).
2) The nested case-control study was conducted based on an established kidney transplant recipients’ cohort with regular blood specimens collected. DSA was detected at least once per year after kidney transplantation. All recipients with the first episode of *de novo* DSA or ABMR within 2 years after kidney transplantation were enrolled, and controls were selected from recipients with stable allograft function and 2 years of follow-up. Patients with pretransplant existing DSA, mixed rejection, previous rejection episode, or any type of infection during the follow-up were excluded. The recipients with stable allograft function met the following criteria during follow-up after recovery: 1) whose urine protein was negative, 2) whose Scr was less than 115 umol/L and undulated within ±20% of the mean Scr, and 3) whose DSA was negative. DSA/ABMR group consisted of 8 ABMR (DSA+ABMR+ group) and 8 DSA-positive patients who refused biopsy because of stable allograft function (DSA+ABMR± group). In the DSA+ABMR+ group, there were 7 recipients also being enrolled in the diagnostic study. De novo DSA (dnDSA) occurred before or concurrent with ABMR. The occurrence of dnDSA and ABMR were set as the endpoints to explore the predictive performance of sBAFF. The specimens of these recipients were collected preoperatively; at day 7, month 1, month 3, month 6, month 9, month 12, and month 18 after transplantation; and at the time of allograft biopsy.

The patients in this cohort were induced with rabbit anti-thymocyte globulin (rATG, Sinofil) or basiliximab (Novartis). rATG was given at a dose of 50 mg during the operation and daily in the following 2 days after transplant. Basiliximab was given at a dose of 20 mg on postoperative days 0 and 4. The maintenance immunosuppressive therapy consisted of tacrolimus in combination with a proliferation inhibitor (mycophenolate mofetil or enteric-coated mycophenolate sodium) and steroids. The target trough level of tacrolimus was 6–10 ng/ml for months 1–3, 6–8 ng/ml for months 4–12, and 5–8 ng/ml thereafter. The dose of mycophenolate mofetil (enteric-coated mycophenolate sodium) was 1,500–2,000 (1,080–1,440) mg/day for weeks 1–4 and 1,000–1,500 (720–1,080) mg/day thereafter.

Written informed consent was obtained from all patients, and the study was approved by the ethics committee of the First Affiliated Hospital of Sun Yat-sen University and conformed to World Medical Association Declaration of Helsinki and the Department of Health and Human Services Belmont Report.

### Serum Soluble B Cell-Activating Factor Detection

Levels of sBAFF in serum samples were measured using Luminex-MAGPIX multiplex immunoassay system according to the manufacturer’s recommended procedures (R&D Systems, Minneapolis, Minnesota, USA) and recorded with the unit of picograms per milliliter (pg/ml). The laboratory staff was blinded to the clinical diagnosis.

### Sample Size

The sample size of the diagnostic study was calculated by formulas (17):


$$V\left(\theta \right)=\frac{\theta }{\text{R}\left(2-\text{θ}\right)}+\frac{2\theta^{2}}{1+\theta }-\theta^{2}\left(\frac{1+R}{R}\right)$$

$$N_{+}=\frac{(Z_{\alpha }\sqrt{V(\theta_{0)}}+Z_{\beta }\sqrt{V{\left(\theta_{1)}\right)}^{2}}}{{\left(\theta_{1}+\theta_{0}\right)}^{2}}$$

where *R* is the ratio of sample size in negative cases and positive cases, *θ_1_* is the predictive area under the receiver operating characteristic (AUROC) curve, while *θ_0_* is the null hypothesis value. *V(θ)* is the variance of *θ* under the null hypothesis of equality. *N_+_* represents the number of positive cases; *α* refers to type I error and *β* to the power of the test. *Z_α_* and *Z_β_* are the boundary values of the standard normal distribution corresponding to parameter (1-*α*) and *β*, respectively.

In our study, we set *R* = 2, *α* = 0.05, *β* = 0.90, and *θ_0_* = 0.5. The *θ_1_* was set at 0.725 according to the literature (18). From the calculation, the number of ABMR cases and non-ABMR cases was 25 and 50, respectively.

### Statistical Analysis

Continuous data following the normal distribution were recorded as the mean ± standard deviation (SD) and were compared by using Student’s t-test or analysis of variance (ANOVA). Continuous data with skew distribution were recorded as the median and interquartile range (IQR) and compared by Mann–Whitney U test or Kruskal–Wallis test. Categorical variable was presented as counts and percentages and compared by the chi-square test or Fisher’s exact test. Correlations were performed by Pearson and Spearman’s rank correlations. The overall comparison between longitudinal groups was performed by linear mixed models. Friedman test and Wilcoxon signed-rank test were used for the comparison of the different time points within the group. ROC, area under the curve (AUC), and Kaplan–Meier curves were used as indexes to evaluate the diagnostic and predictive accuracy of BAFF to ABMR. De Long test was calculated to compare model AUROCs, and log-rank test was used to analyze time-to-event survival curves. Statistical significance was set at *P* < 0.05. Statistical analyses were performed using “R” language (version 4.0.3), a free software environment for statistical computing and graphics (www.r-project.org).

## Results

### Diagnostic Study

**Figure 1:**
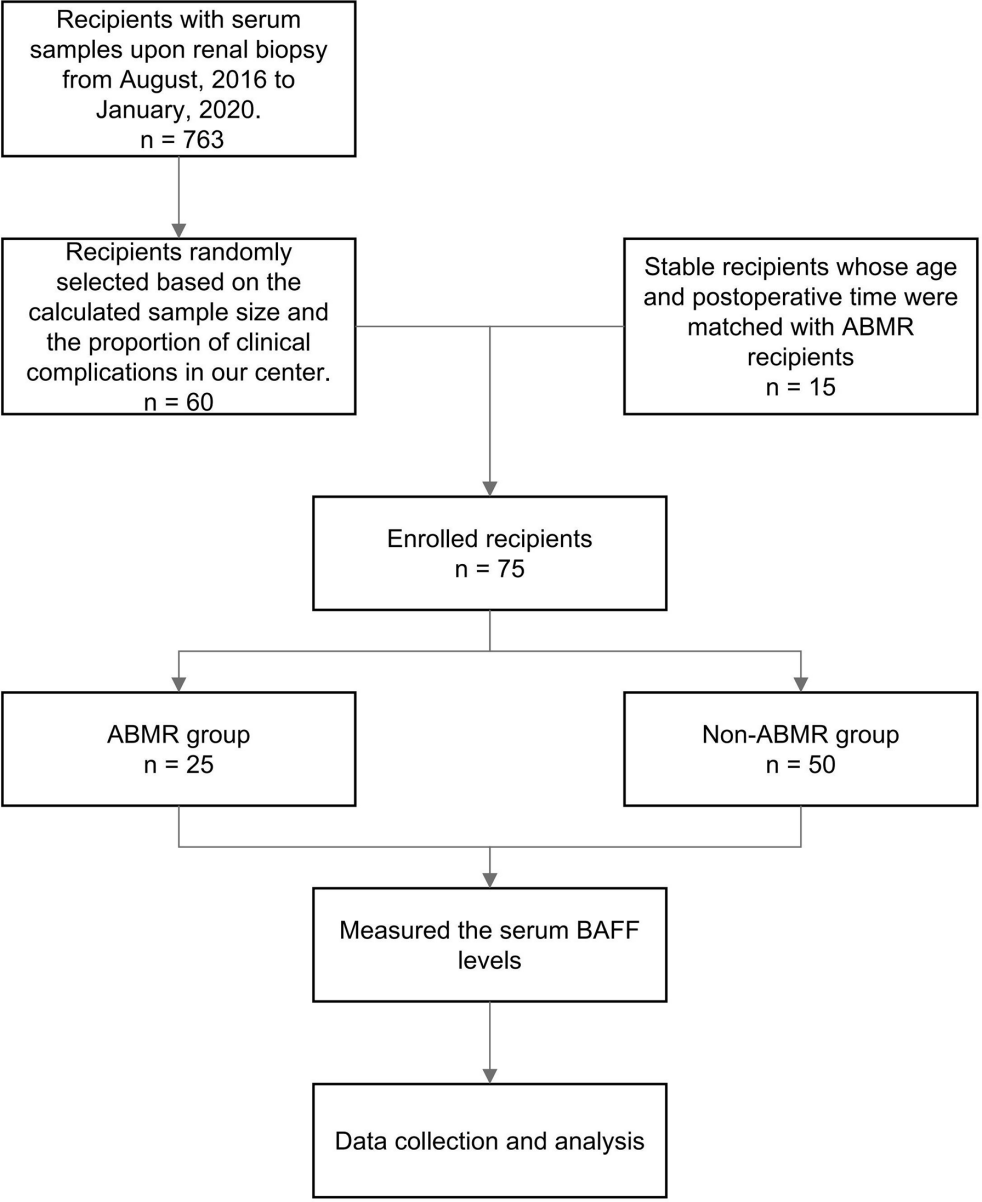
The flowchart of the diagnostic study. ABMR, antibody-mediated rejection; BAFF, B cell-activating factor.

**Figure 2:**
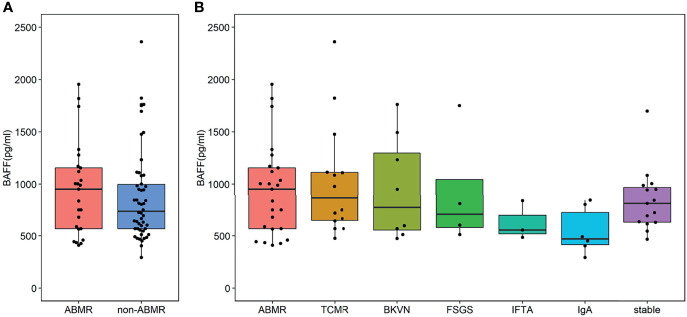
Serum B cell-activating factor (BAFF) levels in kidney transplant recipients. **(A)** The BAFF levels of the antibody-mediated rejection (ABMR) group was comparable to that of the non-ABMR group [951.92 (566.09, 1,162.97) pg/ml vs. 734.77 (564.95, 1,020.04) pg/ml, *P* = 0.438]. The end points of the lower and upper line segments represent 5% and 95%, respectively. The lower and upper horizontal lines of the boxplot mean the first and the third quartile, respectively, while the mid one represents the median. The solid points were outliers (<5th percentile or >95th percentile). **(B)** No significant difference was observed in these groups [951.92 (568.15, 1,155.23) pg/ml vs. 862.32 (647.15, 1112.30) pg/ml vs. 773.09 (554.98, 1,297.68) pg/ml vs. 705.58 (580.18, 1,044.08) pg/ml vs. 55.10 (519.96, 696.14) pg/ml vs. 470.95 (415.30, 723.17) pg/ml vs. 812.46 (633.45, 965.26) pg/ml, *P* = 0.168].

Seventy-five kidney allograft recipients who met the inclusion criteria were enrolled in this study (ABMR 25, BKVN 8; FSGS 4; IFTA 3; IgA 6; TCMR 14, stable 15) (**Table 1** and **Figure 1**). Of 25 patients in the ABMR group, 24 patients had the first rejection episode since transplantation. Only 1 patient had a previous episode about 9 months ago when his pathological findings were suspected to be ABMR while DSA was negative. There were 3 recipients who were HLA class I positive exclusively, 18 recipients were HLA class II positive exclusively, and 4 recipients were positive for both. The serum BAFF level of ABMR was comparable to that of the non-ABMR group [951.92 (566.09, 1,162.97) pg/ml vs. 734.77 (564.95, 1,020.04) pg/ml, *P* = 0.438] (**Figure 2A**). AUROC curve for sBAFF to discriminate ABMR from other pathological lesions was 0.555 (95% CI: 0.408–0.702, *P* = 0.438). There was no significant difference among the seven groups (*P* = 0.168) (**Figure 2B**). The detailed pathology scores within the ABMR groups were shown in **Supplementary Table S1**. There was no relationship found between pathology score and sBAFF level (**Supplementary Figure S1**). Additionally, we classified the ABMR patients according to the max DSA MFI, as follows: low MFI, 1,000–5,000; moderate MFI, 5,000–10,000; and high MFI >10,000. The median MFI was 7,212 (3,423, 15,995), and the distribution of the MFI was shown in **Supplementary Table S2**. No correlation was identified between DSA MFI and sBAFF level (*P* = 0.919, **Supplementary Figure S2**).

**Table 1:** The demographics and clinical characteristics of kidney transplant recipients and donors in the diagnostic study.

Variables	Total (n = 75)	ABMR (n = 25)	Non-ABMR (n = 50)	*P*
Donors				
Age, mean ± SD	36.5 ± 17.9	38.8 ± 20.5	35.1 ± 16.3	0.452
Types, n (%)				0.244
LD	22 (29)	10 (40)	12 (24)	
DD	53 (71)	15 (60)	38 (76)	
Recipients				**0.046**
Gender, n (%)				
Women	25 (33)	4 (16)	21 (42)	
Men	50 (67)	21 (84)	29 (58)	
Age, mean ± SD	38.6 ± 12.8	40.6 ± 14.1	37.6 ± 12.2	0.382
Weight, mean ± SD	55.5 ± 13.0	57.3 ± 9.1	54.7 ± 14.5	0.352
Pretransplantation DSA, n (%)				0.091
Negative	69 (92)	21 (84)	48 (96)	
Positive	6 (8)	4 (16)	2 (4)	
Previous transplantation, n (%)				0.546
0	72 (96)	25 (100)	47 (94)	
1	3 (4)	0 (0)	3 (6)	
Days from transplantation to samples, median (Q1, Q3)	390 (250, 776)	471 (307, 1506)	382 (220, 696)	0.092
Inducement, n (%)				0.283
ATG	58 (77)	17 (68)	41 (82)	
Basiliximab	17 (23)	8 (32)	9 (18)	

LD, living donor; DD, deceased donor; DSA, donor-specific antibody; ABMR, antibody-mediated rejection; ATG, anti-thymocyte globulin. Bold text is used if P value in table is less than 0.05.

### Nested Case-Control Study

**Figure 3:**
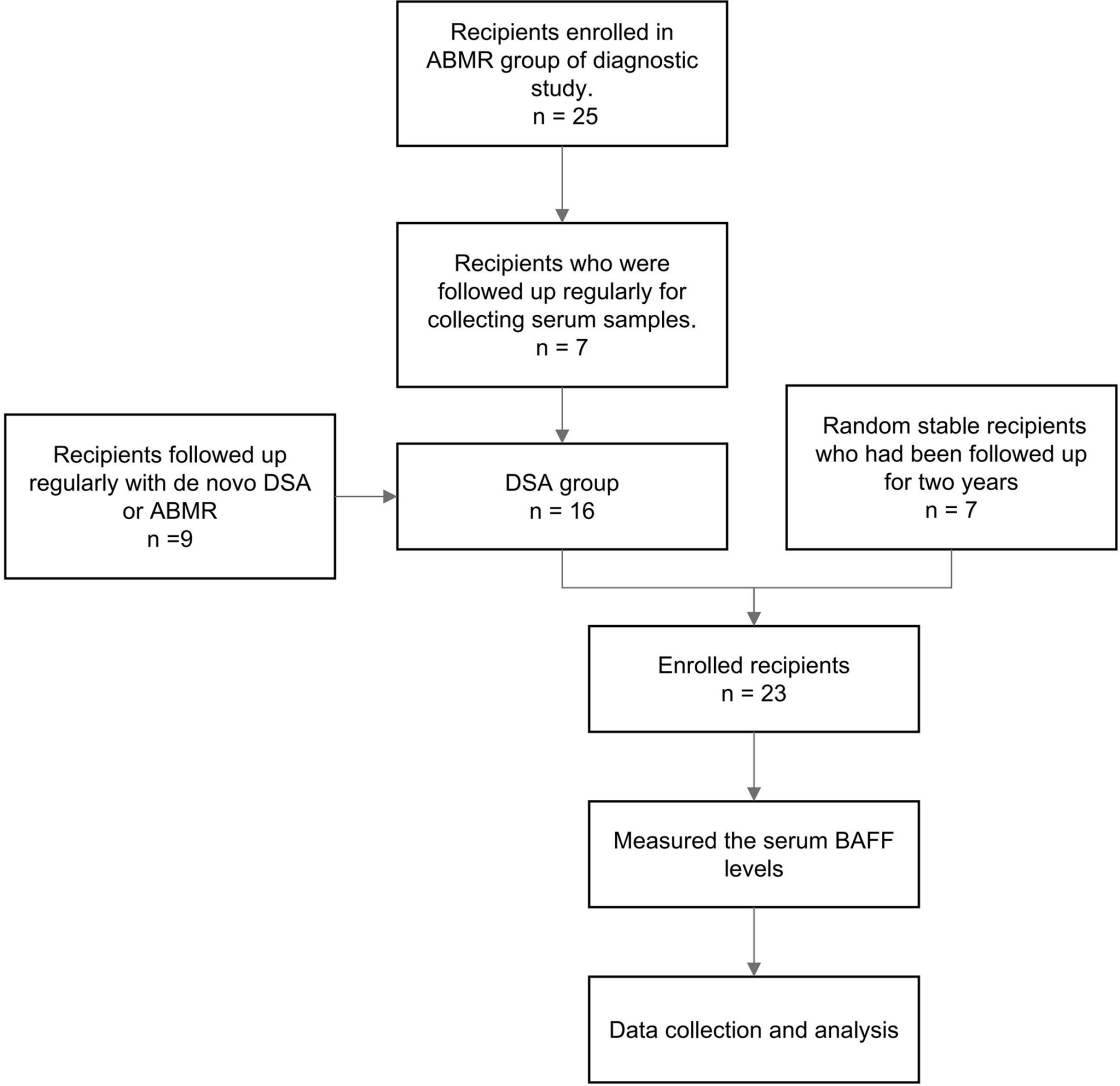
The flowchart of the nested case-control study. ABMR, antibody-mediated rejection; DSA, donor-specific antibody; BAFF, B cell-activating factor.

**Figure 4:**
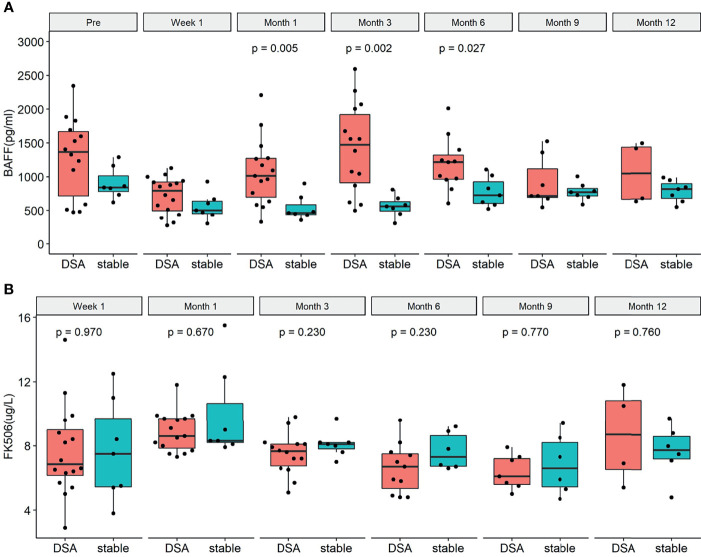
The dynamic changes of serum B cell-activating factor (BAFF) levels and tacrolimus concentration in the donor-specific antibody (DSA) group and the stable group before the occurrence of DSA. **(A)** Dynamic changes of BAFF levels in the DSA group and the stable group. There were significant differences between the two groups at 1 month (*P* = 0.006), 3 months (*P* = 0.003), and 6 months (*P* = 0.033). **(B)** Dynamic changes of tacrolimus concentration in the DSA group and the stable group. There was no significant difference between the two groups at different time points [W1: 6.9 (6.2, 9.0) pg/L vs. 7.5 (5.5, 9.7) pg/L, *P* = 0.947; M1: 8.6 (7.9, 9.7) pg/L vs. 8.3 (8.2, 10.7) pg/L pg/L, *P* = 0.670; M3: 7.7 (6.8, 8.1) pg/L vs. 8.1 (7.8, 8.2) pg/L, *P* = 0.230; M6: 6.7 (5.4, 7.5) pg/L vs. 7.3 (6.7, 8.6) pg/L, *P* = 0.230; M9: 6.1 (5.6, 7.2) pg/L vs. 6.6 (5.5, 8.2) pg/L, *P* = 0.770; M12: 6.2 (4.4, 8.1) pg/L vs. 7.8 (7.2, 8.6) pg/L, *P* = 0.480; respectively].

A total of 16 patients in the longitudinal cohort tested positive for DSA (including 8 recipients with ABMR and 8 recipients with DSA positivity but no biopsy result, **Figure 3**). The demographics and clinical characteristics of the DSA group and stable group were comparable except recipient age (**Table 2**). The detailed pathology scores of ABMR patients in the nested case-control study were shown in **Supplementary Table 3**. The overall dynamic changes of sBAFF level in the DSA+ABMR+ group, DSA+ABMR± group, and stable group were shown in **Supplementary Figure S3**. The trends of the DSA+ABMR+ group and DSA+ABMR± group were similar, and there was no significant difference between these two groups at any time point. So, we combined these two groups as DSA group. There were 16 recipients who were HLA class II positive exclusively, 1 recipient was positive for both, and no recipient was HLA class I positive exclusively. The median max DSA MFI of ABMR was 3,987 (1,498, 7,667), and the distribution of the MFI was shown in **Supplementary Table S4**. Dynamic changes of BAFF levels were compared between the DSA group and the stable group (**Figure 4A**). The sBAFF levels of stable patients significantly differed at different time points [840.92 (782.82, 1,012.53) pg/ml vs. 501.73 (453.34, 637.04) pg/ml vs. 462.69 (438.77, 586.48) pg/ml vs. 561.63 (489.77, 630.00) pg/ml vs. 726.93 (604.77, 924.60) pg/ml vs. 772.33 (716.46, 828.34) pg/ml vs. 812 (676.87, 894.24) pg/ml, *P* < 0.001]. The sBAFF levels decreased significantly during the first week after surgery (*P* = 0.018) and maintained at a low level within 3 months after transplantation. Subsequently, there was a slow increase within 3–6 months (*P* = 0.018) and then maintained at a plateau.

**Table 2:** The demographics and clinical characteristics of DSA group and stable group in the nested case-control study.

Variables	Total (n = 23)	DSA (n = 16)	Stable (n = 7)	*P*
Donors				
Age, mean ± SD	31.09 ± 20.43	30.75 ± 23.37	31.86 ± 12.81	0.886
Type, n (%)				1
DD	16 (70)	11 (69)	5 (71)	
LD	7 (30)	5 (31)	2 (29)	
Recipients				
Gender, n (%)				0.182
Women	8 (35)	4 (25)	4 (57)	
Men	15 (65)	12 (75)	3 (43)	
Age, mean ± SD	31.46 ± 15.15	26.84 ± 14.55	42.01 ± 11.2	**0.016**
Weight, median (Q1, Q3)	53 (47.5, 62.25)	56.5 (46.38, 63)	53 (50, 56.5)	0.893
Pretransplantation DSA, n (%)				1
Negative	23 (100)	16 (100)	7 (100)	
Previous transplantation, n (%)				1
0	23 (100)	16 (100)	7 (100)	
Induce, n (%)				1
ATG	19 (83)	13 (81)	6 (86)	
Basiliximab	4 (17)	3 (19)	1 (14)	

LD, living donor; DD, deceased donor; DSA, donor-specific antibody; ATG, anti-thymocyte globulin.

In the DSA group, the median time from transplantation to DSA positivity test was 328 (206, 420) days. Dynamic changes of sBAFF levels of each recipient were shown in the Supplementary Material (**Supplementary Figure S4**). Within the first postoperative week, the sBAFF levels were notably decreased [1,367.57 (569.27, 1,726.94) pg/ml vs. 691.92 (423.54, 912.52) pg/ml, *P* = 0.001], whose trend was similar to it in the stable group. However, the serum BAFF levels of the DSA group significantly increased from week 1 to month 1 [854.30 (511.45, 938.54) pg/ml vs. 1,013.23 (633.97, 1,277.38) pg/ml, *P* = 0.006] and reached a peak at month 3, followed by a downward trend. The serum sBAFF levels in DSA group were significantly increased compared with the stable group at 1 month [1,013.23 (633.97, 1,277.38) pg/ml vs. 462.69 (438.77, 586.48) pg/ml, *P* = 0.007], 3 months [1,472.07 (912.79, 1,922.08) pg/ml vs. 561.63 (489.77, 630.00) pg/ml, *P* = 0.002], and 6 months [1,217.95 (965.25, 1,321.43) pg/ml vs. 726.93 (604.77, 924.60) pg/ml, *P* = 0.027]. Meanwhile, the tacrolimus concentrations of the two groups were consistently comparable at different time points (**Figure 4B**).

**Figure 5:**
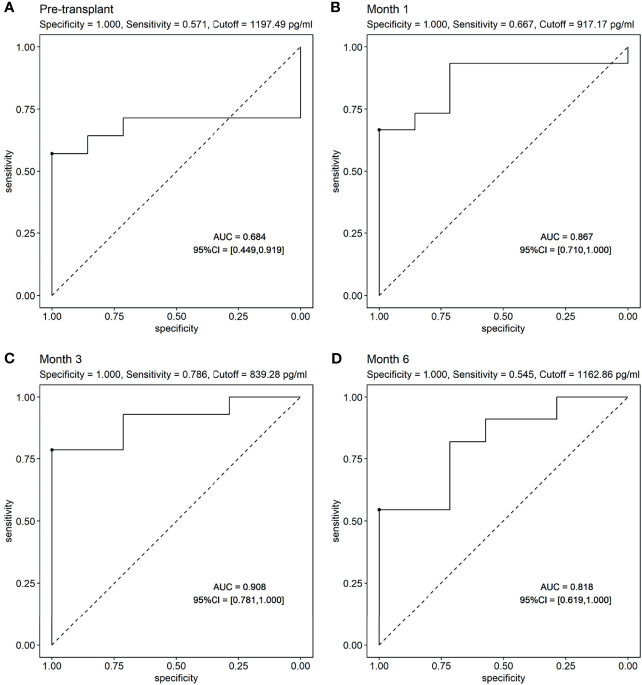
The receiver operating characteristic (ROC) curves of soluble B cell-activating factor (sBAFF) levels at different time points in predicting the donor specific antibody (DSA). **(A)** The ROC curve of pretransplant sBAFF levels to predict the dnDSA. The pretransplant sBAFF levels had no predictive value for dnDSA. **(B–D)** The ROC curve of sBAFF levels at M1, M3 and M6 to predict the dnDSA. The sBAFF levels at M3 had the biggest AUC (0.908), with a good sensitivity (0.786) and specificity when the threshold was 839.28 pg/ml.

**Figure 6:**
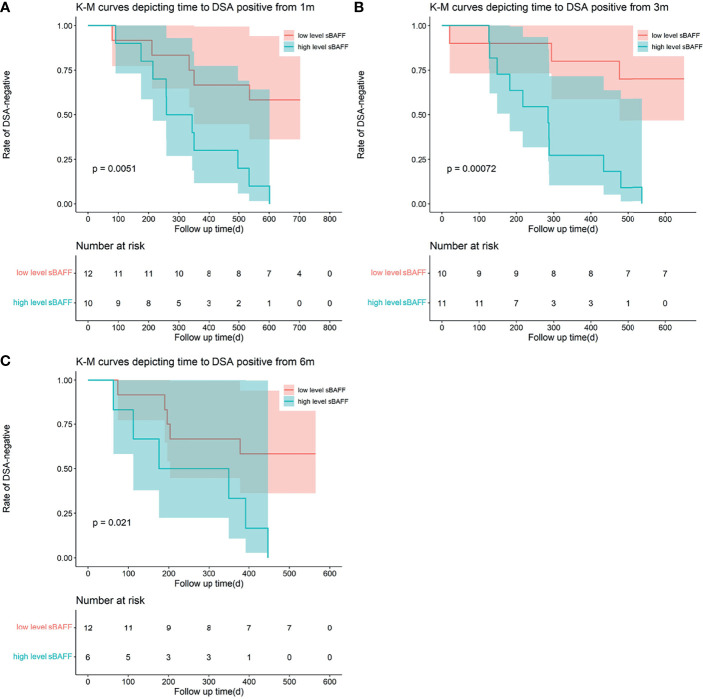
Kaplan–Meier curves depicting time to the occurrence of dnDSA from 1 month (m), 3 m, and 6 m after transplantation. The recipients were divided into two groups according to the cutoff calculated by the receiver operating characteristic (ROC). **(A–C)** High-level soluble B cell-activating factor (sBAFF) at 1 m, 3 m, and 6 m was a risk factor for the occurrence of the dnDSA.

ROC curves were used to show the performance of the sBAFF levels preoperatively and at months 1, 3, and 6 after transplantation in predicting the DSA (**Figure 5**). The third month after the surgery showed the highest AUC (0.908, 95% CI: 0.781–1.000), with satisfactory sensitivity (78.6%) and specificity (100.0%) using the cutoff value of 839.28 pg/ml. Patients in the DSA group had DSA positivity after a median of 286 (125, 399) days from month 3. According to the cutoff of months 1, 3, and 6 (cutoff = 917.17, 839.28, and 1,162.86 pg/ml, respectively), all recipients were divided into two groups. At months 1, 3, and 6, high-level sBAFF levels increased the risk of *de novo* DSA (**Table 3** and **Figure 6**). Although the difference in pretransplant sBAFF level was not statistically significant between the DSA group and the stable group (*P* = 0.179), pretransplant sBAFF level was moderately correlated with month 3 sBAFF level (r = 0.662, *P* = 0.002). To investigate the diagnostic value of sBAFF change rate before *de novo* DSA occurrence for the occurrence of *de novo* DSA, sBAFF levels were compared before and at the occurrence of *de novo* DSA. No significant difference of sBAFF levels was observed [before vs. during DSA positivity: 957.60 (676.04, 1,385.03) pg/ml vs. 860.06 (678.10, 1,011.31) pg/ml, *P* = 0.307]. The median of difference calculated by subtracting the value of the previous time point from the latter was -22.65 (-400.03, 146.78) pg/ml. The median of interval time was 86 (80, 120) days.

**Table 3:** Risk for *de novo* DSA after kidney transplantation (KTx) in high-level sBAFF group vs. low-level sBAFF group at different time points.

Time Points	Groups	HR	95% CI	*P*
Month 1	>900 vs. ≤900	4.791	1.479–15.518	0.009
Month 3	>850 vs. ≤850	7.067	1.894–26.370	0.004
Month 6	>1,200 vs. ≤1,200	3.664	1.103–12.168	0.034

DSA, donor-specific antibody; sBAFF, soluble B cell-activating factor; HR, hazard ratio; KTx, kidney transplant.

**Figure 7:**
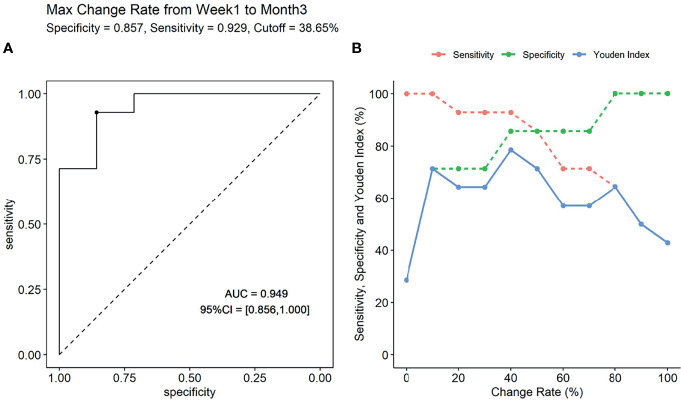
The predictive performance of max change rate from the first week to the third month for the occurrence of donor-specific antibody (DSA). **(A)** The receiver operating characteristic (ROC) curve (if the max change rate is 38.65%, the Youden index is maximal). **(B)** The sensitivity, specificity, and Youden index were presented according to specific change rate threshold.

We also explored the predictive performance of dynamic change within 3 months for *de novo* DSA occurring after that. Change rate of sBAFF levels was obtained by subtracting the values of two time points and dividing the difference by the value of the previous time point. Two recipients in the DSA group were detected to be DSA positive within the third month; thus, they were removed from this analysis. The max sBAFF change rate within 3 months after transplantation, calculated by values at the first week and the maximum values at the first or third month [max change rate = (the maximum values at the first/third month - the first week)/the first week], was first evaluated. The maximum sBAFF change rate of the DSA group was significantly higher compared with that of the stable group [90.0% (63.1%, 143.8%) vs. 3.2% (-0.7%, 19.7%), *P* = 0.001]. The AUC of the max change rate for predicting *de novo* DSA was 0.949 (95% CI: 0.856–1.000), higher than the AUC of sBAFF level at month 3, although the difference was not significant (*P* = 0.580). The cutoff value that maximized sensitivity at 92.9% and specificity at 85.7% was 38.6% (**Figure 7**). The joint predictive value of the maximum sBAFF change rate within 3 months and the absolute quantification of sBAFF levels at the third month was also high (AUC = 0.949, 95% CI: 0.860–1.000). Taking the clinical use into consideration, we set the threshold of absolute quantification on the third month postoperatively at the value of 850 pg/ml and threshold of max change rate from the first week to the third month at the value of 40%. The distribution of the recipients according to the threshold was shown in **Table 4**.

**Table 4:** The distribution of the recipients in 4 groups based on different sBAFF levels.

Group	DSA/ABMR, n (%)	Stable, n (%)
Total	14 (100%)	7 (100%)
Quantification ≥850 pg/ml and max change rate ≥40%	10 (71.4%)	0 (0%)
Quantification ≥850 pg/ml but max change rate <40%	1 (7.1%)	0 (0%)
Max change rate ≥40% but quantification <850 pg/ml	3 (21.4%)	1 (14.3%)
Neither quantification ≥850 pg/ml or change rate ≥40%	0 (0%)	6 (85.7%)

sBAFF, soluble B cell-activating factor.

We also evaluated the predictive value of the change rate from week 1 to month 3 for *de novo* DSA. From week 1 to month 3, 13 of the 14 recipients in the DSA group have an increase in sBAFF levels, while only three recipients in the stable group had elevated sBAFF levels (*P* = 0.025). There was a significant difference in the change rate from week 1 to month 3 between these two groups [90% (53%, 157%) vs. -4% (-14%, 19%), *P* = 0.005]. The AUC of the change rate was 0.888 (95% CI: 0.741–1.000), and the cutoff value that maximized sensitivity at 85.7% and specificity at 85.7% is 38.65%.

Of the 16 patients with DSA positivity, ABMR was confirmed in 8 cases. To investigate the diagnostic value of sBAFF change rate before the biopsy for the ABMR, sBAFF levels were compared before and at the biopsy. No significant difference of sBAFF levels was observed before and at biopsy [987.27 (874.31, 1,119.65) pg/ml vs. 989.54 (660.23, 1,019.69) pg/ml, *P* = 0.612]. The median of the difference obtained by subtracting the value before biopsy from the value at biopsy was -88.74 (-185.48, 78.74) pg/ml, and the median of interval time was 86 (58, 107) days. The sBAFF levels of these ABMR patients showed an upward trend from the first month to the third after surgery, earlier than that of the stable group (**Supplementary Figure S5**). The ABMR group had higher levels of serum BAFF than those of the stable group at the third month after surgery (*P* = 0.029). The AUC of the sBAFF levels at the third month to predict ABMR was 0.839 (95% CI: 0.621–1.000), and the optimal threshold was 839.28 pg/ml. The ABMR group had higher max change rate from the first week to the third month than that of the stable group. There was a significant difference existing in the max change rate from the first week to the third month [67.5% (44.5%, 93.5%) vs. 3% (-0.5%, 19.5), *P* = 0.049]. The AUC of max change rate to predict ABMR was 0.804 (95% CI: 0.557–1.000), with an optimal cutoff value of 45.16%, at which the sum of the sensitivity and specificity was maximal.

## Discussion

This study is to evaluate the diagnostic value of sBAFF for ABMR in kidney allograft by a diagnostic test design, and meanwhile, we also investigated the predictive value of early sBAFF monitoring for postoperative *de novo* DSA risk after renal transplantation by intensive early postoperative sampling and a nested case-control design. The diagnostic test showed that absolute sBAFF value was not diagnostic of ABMR (AUROC = 0.633, *P* = 0.125). sBAFF did not change significantly before the onset of ABMR vs. at the time of diagnosis of ABMR, suggesting that the change in sBAFF values before the onset of ABMR was also not diagnostic of renal transplant ABMR. The nested case-control study demonstrated that in low-risk recipients without preoperative DSA, sBAFF values at M3 posttransplant and the max change rate from D7 to the peak within M3 had a good predictive value for the risk of DSA/ABMR occurrence within 2 years after renal transplantation (AUROC 0.908 and 0.949, respectively), with the performance much better than tacrolimus trough concentration. We found by a previous meta-analysis that preoperative or postoperative single-point sBAFF values were predictive of ABMR after kidney transplantation. In the present study, we found that early postoperative dynamic monitoring of changes in sBAFF levels was probably a better predictor of *de novo* DSA risk than absolute sBAFF values at any single time point preoperatively or postoperatively. This provides a useful tool for early screening of low-risk patients with negative preoperative DSA for the risk of developing postoperative *de novo* DSA.

It was noted that pretransplant sBAFF was higher in the dnDSA/ABMR group compared with that in the stable group, although the difference was insignificant due to the small sample size. The meta-analysis we performed previously indicated that pretransplant sBAFF level was associated with ABMR occurrence after kidney transplantation (14). Besides, pretransplant sBAFF level was moderately correlated with month 3 sBAFF level (r = 0.662, *P* = 0.002), which was a robust index of dnDSA/ABMR risk in our study. These indicated the potential predictive value of pretransplant sBAFF level.

Our longitudinal cohort completely described the dynamic changes of sBAFF levels for kidney transplantation recipients within the first year. Comoli et al. (16) found that sBAFF levels would continue to increase within the first year of kidney transplantation and stabilize thereafter. Their finding was broadly similar to the dynamic changes of our stable group. However, in their study, the early decline and trough period were lacking because the monitoring points from preoperative to the first 3 months after surgery were missing. In the study by Comoli et al. (16), they demonstrated that sBAFF monitoring could not predict the development of the DSA. Conversely, in our cohort, the sBAFF levels at months 1, 3, and 6 have a good predictive ability for DSA. The difference might be caused by that the DSA of our cohort occurred earlier than theirs. In their cohort, DSA appeared at a median of 24 months after transplantation, ranging from 3 to 93 months. Schuster et al. (15) also performed a cohort study on the association of sBAFF level and DSA development after kidney transplantation and longitudinally detected sBAFF level at 14 days, 3 months, and 12 months posttransplant. In their cohort, sBAFF level was low at day 14 and kept increasing within 1 year, also similar to that of the stable group in our cohort. Due to lack of pretransplant detection of sBAFF, they failed to catch the early decline of sBAFF. Moreover, they found that higher sBAFF level at day 14 was associated with the increased risk of rejection, especially intimal arteritis and peritubular capillaritis, and that posttransplant sBAFF level was correlated with pretransplant immunologic risk that was based on DSA status, Complement-dependent cytotoxicity (CDC) examination, and previous transplant history. Unfortunately, they failed to confirm the independent predictive effect of sBAFF on DSA/ABMR risk, while the present study demonstrated the independent predictive value of sBAFF on dnDSA/ABMR risk in the low-risk patients with no pretransplant existing DSA, who are in urgent demand for a non-invasive tool for the risk stratification. The significant decrease of all groups within the first week after surgery might result from potent induction therapy and reflect the severely suppressed immune status of the recipients. With the reconstruction of the immune system, the sBAFF levels increased gradually. The early elevation in the DSA group reflected the early activation of the innate immune system, which is the major source of the BAFF (8). It had been proven that, for example, monocyte, dendritic cells, and natural killer (NK) cells could produce BAFF under interleukin-2 (IL-2) stimulation (19). The role of the innate immune system in the ABMR had been focused on. It had been reported that the innate immune system might contribute to the DSA and ABMR, especially early development of active ABMR (20, 21). We speculated that early activation of the innate immune system in the DSA group led to the generation of BAFF. High-level sBAFF activated B cells and prompted the development of DSA. The sBAFF levels may be a potential marker for the active status of the innate immune system. Early intensive postoperative monitoring of sBAFF levels will result in early identification of recipients with a high risk of developing ABMR and early intervention consequently.

In the past, few studies focused on the diagnostic value of BAFF for ABMR. Wang et al. (18) carried out a prospective research aiming to explore the relationship between acute rejection and BAFF, enrolling 155 recipients in the stable group, as well as the acute rejection group consisting of 5 ABMR and 29 TCMR patients. Their results showed that compared with the stable group, the BAFF levels in the acute rejection group was significantly higher when rejection occurred, which contradict our result that there was no significant difference between the ABMR group, TCMR group, and stable group. However, the limited number of ABMR patients in the study by Wang et al. made it difficult to evaluate the relationship between ABMR and BAFF levels in their cohort. Moreover, we enrolled recipients with 4 other pathological lesions except rejection, such as BKVN, FSGS, IFTA, and IgAN, closer to the real clinical situation.

The discrepancy between the presence of predictive value and the absence of diagnostic value of sBAFF in ABMR after kidney transplantation is interesting. An experiment using non-human primate model showed that the sBAFF levels differed along with the development and progression of ABMR. The serum sBAFF level increased within the first week after kidney transplantation when no DSA was detected. However, along with the appearance of alloantibodies and development of ABMR, the serum sBAFF levels showed a decreased trend and even fell back to baseline levels in the late phase (22). The dynamic change of sBAFF in this animal study is similar to the results in the DSA group in our study. The sBAFF levels reached the peak before the detection of DSA and decreased with the production of DSA. This can partly explain the phenomenon that the sBAFF level at the time of ABMR diagnosis, often in the late phase, was comparable to that of the stable recipients. Thibault-Espitia et al. (11) found that the BAFF-R transcript level in peripheral blood mononuclear cell (PBMC) was inversely correlated with the serum sBAFF level. We supposed that the proliferation of B cells and the increased expression of receptors on their surface increased the consumption of the sBAFF in the serum, resulting in the decrease of sBAFF level in the late phase of ABMR (23).

Inevitably, our study had a few limitations. First, this is a single-center diagnostic study; however, the sample size based on the statistical calculation was large enough to provide enough statistical power. Besides, this study was unable to examine the correlation between early sBAFF monitoring and the development of DSA/ABMR in the late postoperative period or the correlation between monitoring of sBAFF in the late postoperative period on the risk of DSA/ABMR development.

In conclusion, we clarified by a diagnostic study that sBAFF is not a diagnostic biomarker for ABMR in kidney transplantation and revealed by a nested case-control study that sBAFF values at 3 months posttransplant and dynamic changes in sBAFF within 3 months posttransplant have a good predictive value for the occurrence of DSA/ABMR within 2 years after kidney transplantation. The latter conclusion still needs to be further clarified by large-scale prospective studies. It is worth mentioning that attention should be paid to the effect of autoimmune disease and infection in further clinical practice. The role of sBAFF in the pathogenesis of ABMR needs to be further explored.

## Data Availability Statement

The raw data supporting the conclusions of this article will be made available by the authors without undue reservation.

## Ethics Statement

The studies involving human participants were reviewed and approved by the Ethics Committee of the First Affiliated Hospital of Sun Yat-sen University. Written informed consent to participate in this study was provided by the participants’ legal guardian/next of kin.

## Author Contributions

SW, HZ, and LL designed the study. SW, XS, and YG analyzed the data and co-wrote the article. MH, YC, QF, and JL enrolled patients and collected the data. QY, YW, CLW, and QZ performed the experiments. JW, HH, and BX collected and cleaned the data. HZ, LL, and CXW supervised the research and critically reviewed the paper. All authors contributed to the article and approved the submitted version.

## Funding

This study was supported by the National Natural Science Foundation of China (81870511, 81670680, 82170770, 31800758, 81700655), Science and Technology Planning Project of Guangdong Province, China (2015B020226002, 2017A020215012), Key Scientific and Technological Program of Guangzhou City (201903010058, 201803040011), Guangdong Provincial Key Laboratory on Organ Donation and Transplant Immunology (2017B030314018, 2020B1212060026), and Guangdong Provincial International Cooperation Base of Science and Technology (Organ Transplantation, 2020A0505020003).

## Conflict of Interest

The authors declare that the research was conducted in the absence of any commercial or financial relationships that could be construed as a potential conflict of interest.

## Publisher’s Note

All claims expressed in this article are solely those of the authors and do not necessarily represent those of their affiliated organizations, or those of the publisher, the editors and the reviewers. Any product that may be evaluated in this article, or claim that may be made by its manufacturer, is not guaranteed or endorsed by the publisher.

## Abbreviations

ABMR, antibody-mediated rejection; ATG, anti-thymocyte globulin; AUCs, areas under the curve; BAFF, B cell-activating factor; BAFFR, B cell-activating factor receptor; BCMA, B-cell maturation antigen; BKV, BK polyomavirus; BKVN, BK polyomavirus nephropathy; CDC, Complement-dependent cytotoxicity; CMV, cytomegalovirus; DD, deceased donor; dnDSA, de novo DSA; DSA, donor-specific antibody; FSGS, focal segmental glomerulosclerosis; HBV, hepatitis B virus; HCV, hepatitis B virus; HR, hazard ratio; IFTA, interstitial fibrosis and tubular atrophy; IQR, interquartile range; KTx, kidney transplantation; LD, living donor; max, maximum;NK cell, natural killer cell; PBMC, peripheral blood mononuclear cell;ROC, receiver operating characteristic; sBAFF, soluble B cell-activating factor receptor; Scr, serum creatinine; SD, standard deviation; TACI, transmembrane activator and calcium modulator and cyclophilin ligand interactor; TCMR, T cellmediated rejection.
