# Supplementary material for: Serum Soluble B Cell-Activating Factor Is a Non-Invasive Biomarker of Antibody-Mediated Rejection in Kidney Allograft With Satisfactory Risk Stratification Performance But Negligible Diagnostic Value
Source: Front Immunol. 2022 Apr 13;13:869444. doi: 10.3389/fimmu.2022.869444 (PMC9045738; doi:10.3389/fimmu.2022.869444)
Supplement: Supplementary file 1 [file DataSheet_1.docx]

**Supplementary Material:**

**
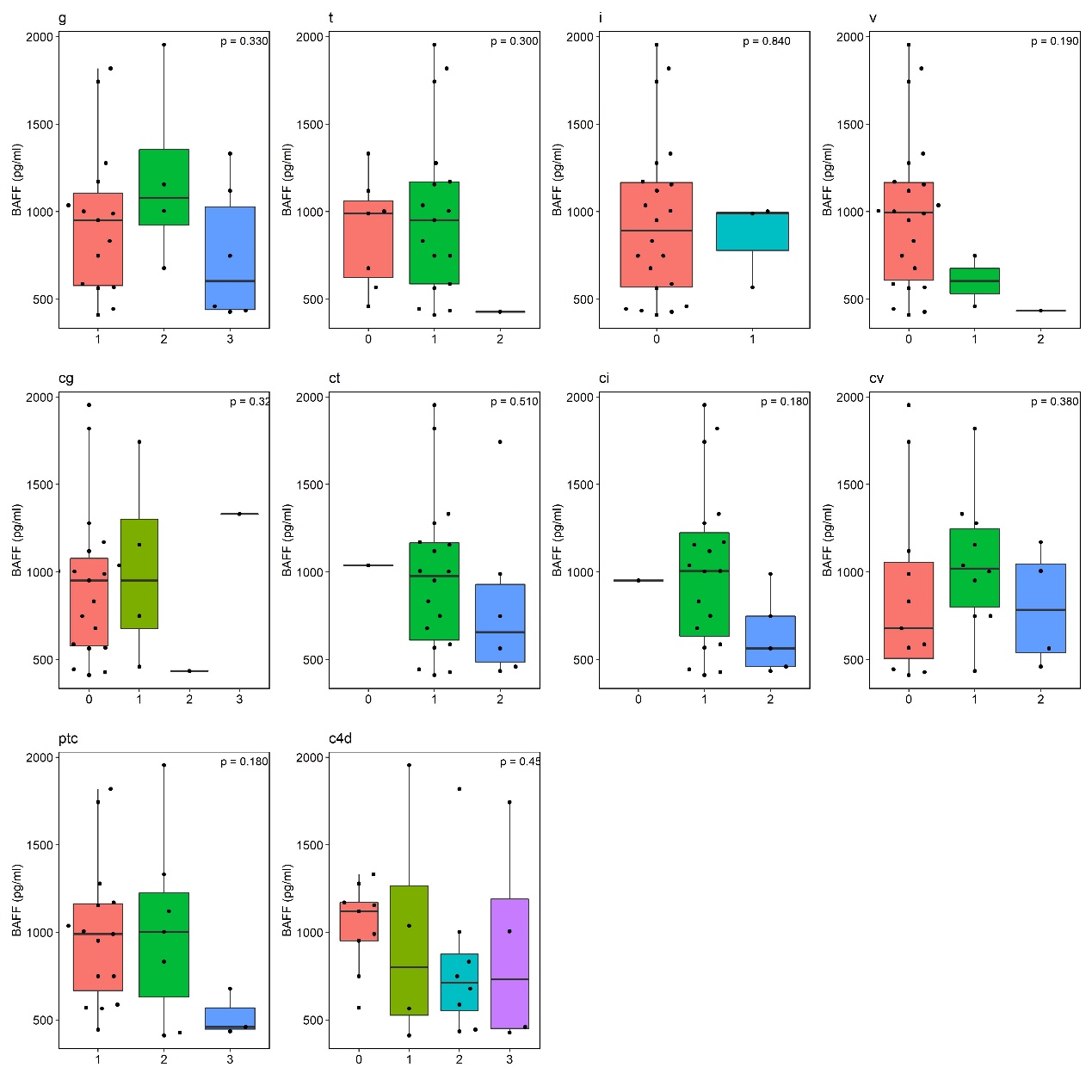
**

**Figure S1|** Correlation between sBAFF level and grades of Banff pathologic categories in ABMR recipients. g, glomerulitis; t, tubulitis; i, inflammation; v, intimal arteritis; cg, glomerular double contours; ct, tubular atrophy; ci: Interstitial fibrosis; cv, vascular fibrous intimal thickening; ptc, peritubular capillary.

**
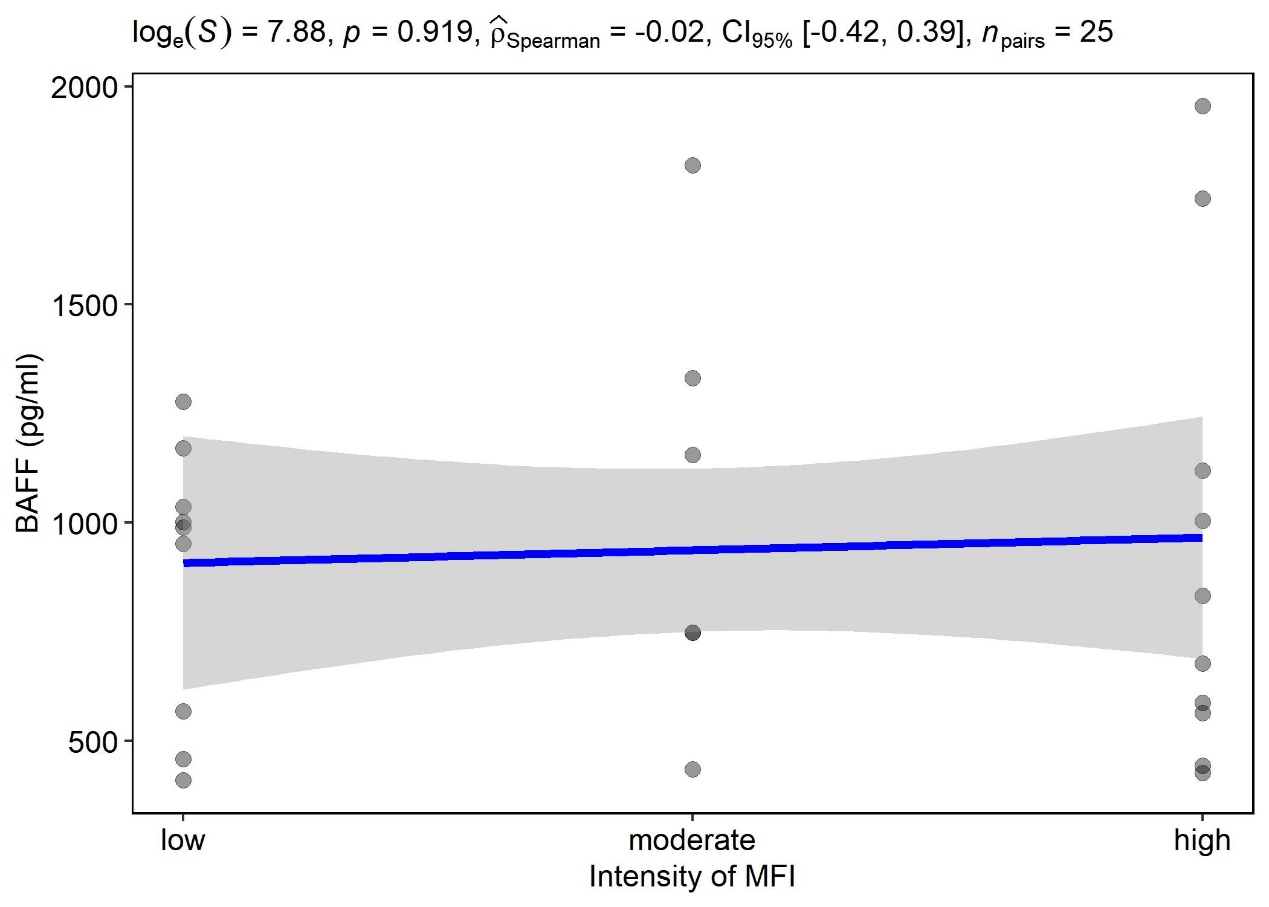
**

**Figure S2|** Relationship between intensity of MFI and sBAFF levels in the diagnostic study.

**
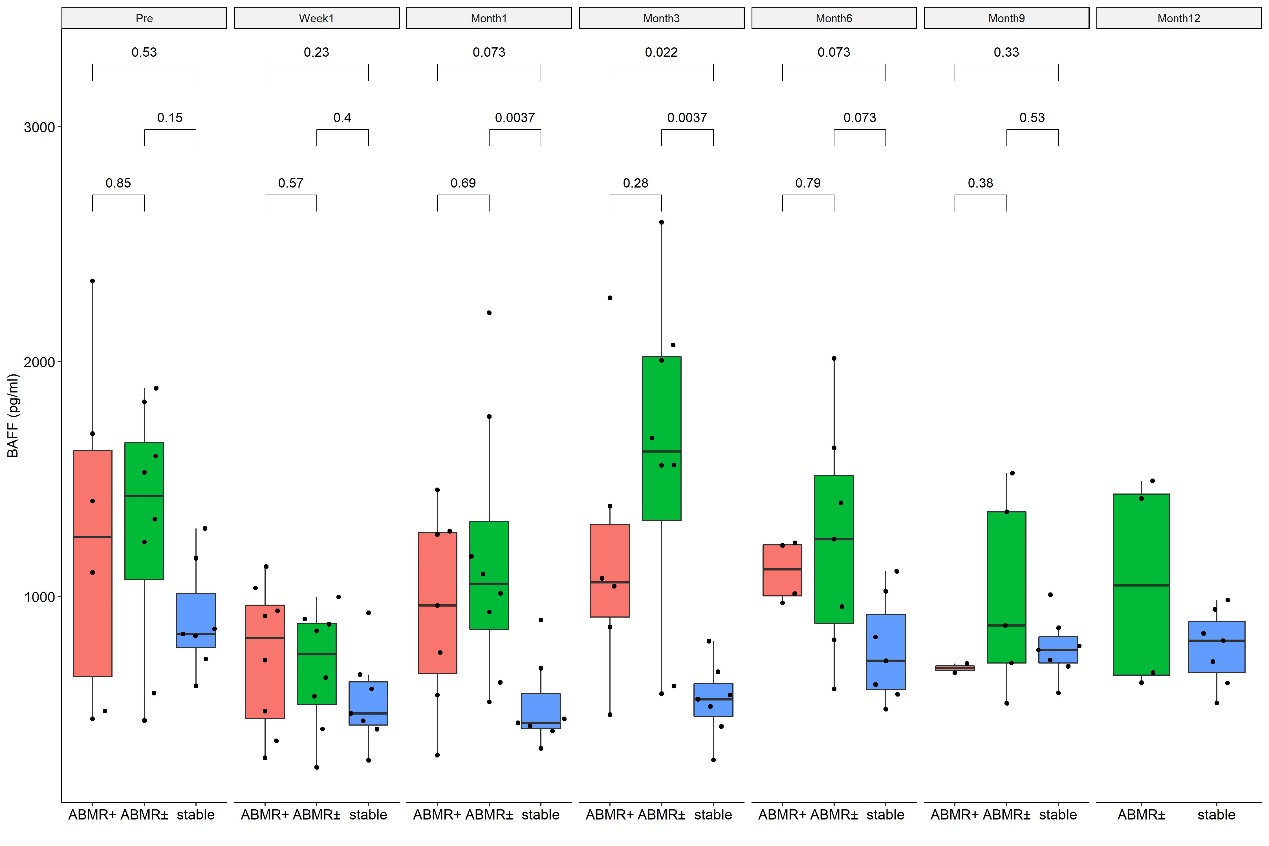
**

**Figure S3|** Dynamic changes of BAFF levels in DSA+ABMR+ group, DSA+ABMR± group and stable group before the occurrence of DSA. There was no significantly difference between the DSA+ABMR+ group and DSA+ABMR± group at any time point.


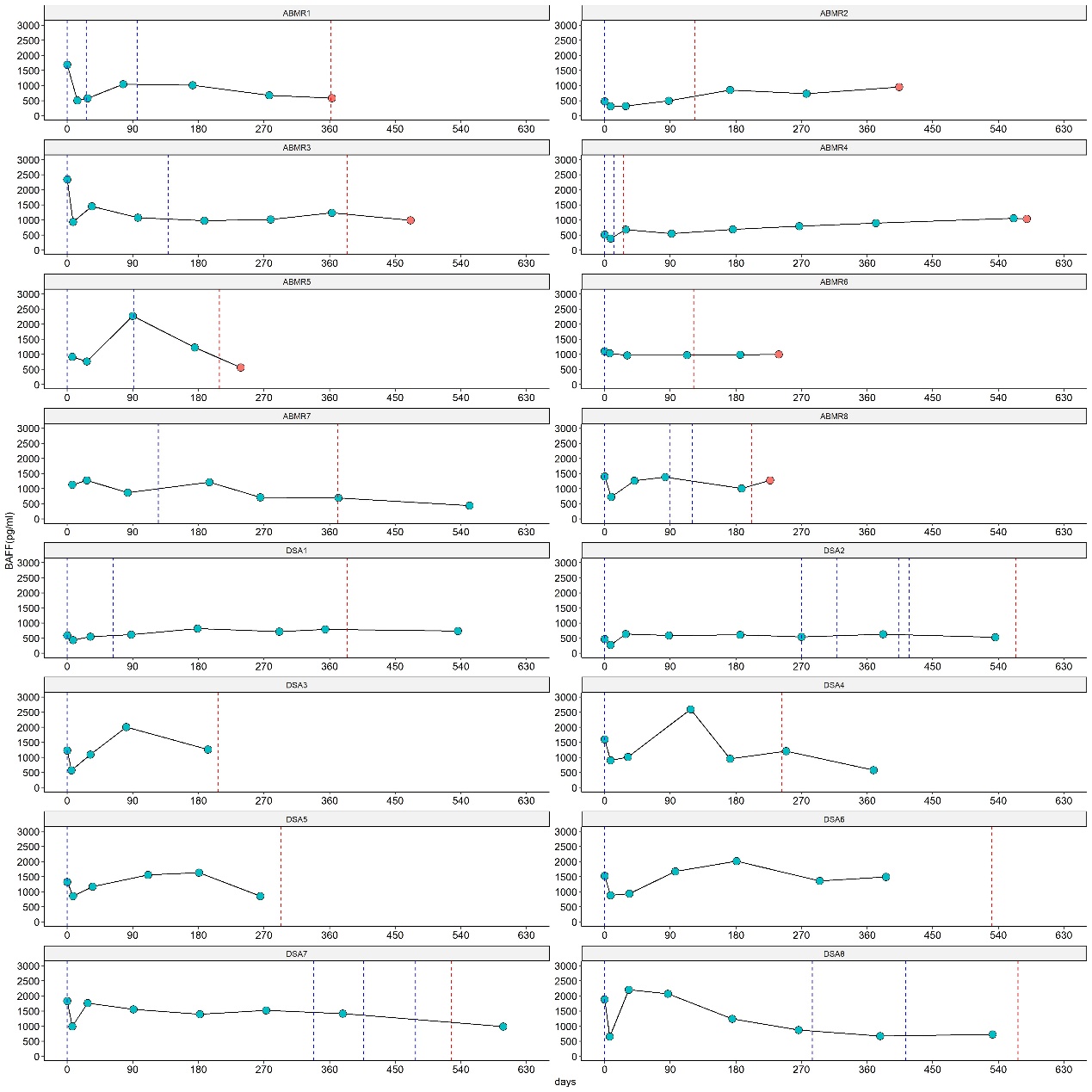


**Figure S4|** the dynamic changes of sBAFF levels of each recipient in DSA/ABMR group. The blue dots signified the sBAFF levels corresponding with each time point. The red dots represented the time point of biopsy and the sBAFF levels corresponding to that. The dashed line showed the status of DSA. Red indicated DSA positive, whereas blue indicated negative. For patient ABMR7, he was proven ABMR by biopsy at 645 days after transplantation. But his preserved specimens at the time of biopsy were run out.


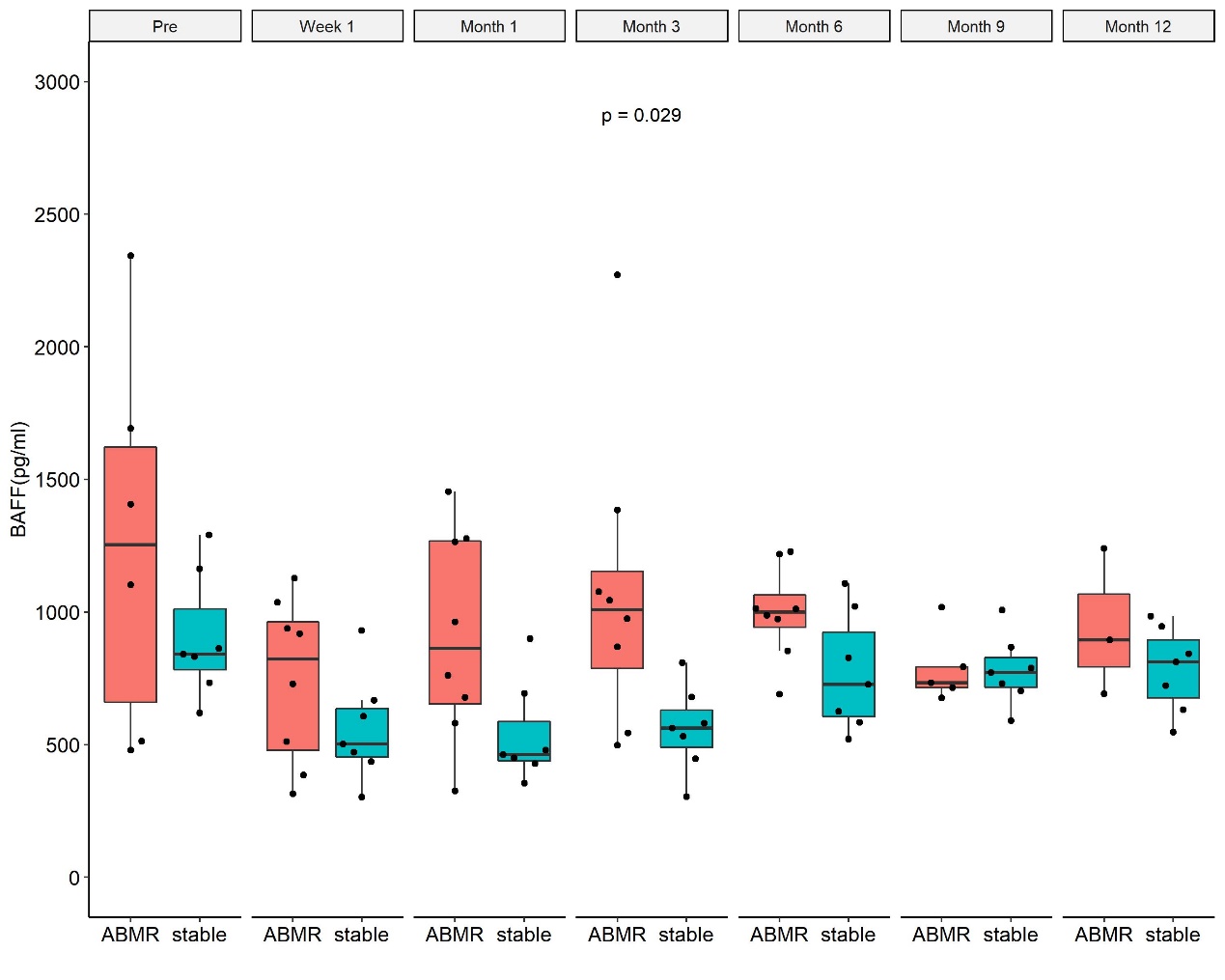


**Figure S5|** Dynamic changes of BAFF levels in ABMR group and stable group before biopsy. There were significantly difference between the two groups at 3 months (921.90 [594.71, 1068.59] vs. 561.63 [489.77, 630.00] pg/ml, P = 0.029).

**TABLE S1|** The lesion score of ABMR patients in the diagnostic study.

| Lesion score | g,  n (%) | ptc  n (%) | t  n (%) | i  n (%) | v  n (%) | cg  n (%) | ct  n (%) | ci  n (%) | cv  n (%) | C4d  n (%) |
| --- | --- | --- | --- | --- | --- | --- | --- | --- | --- | --- |
| 0 | 0 (0) | 0 (0) | 7 (28) | 22 (88) | 22 (88) | 19 (76) | 0 (0) | 0 (0) | 11 (44) | 9 (36) |
| 1 | 15 (60) | 15 (60) | 17 (68) | 3 (12) | 2 (8) | 4 (16) | 18 (72) | 19 (76) | 10 (40) | 4 (16) |
| 2 | 4 (16) | 7 (28) | 1 (4) | 0 (0) | 1 (4) | 1 (4) | 6 (24) | 5 (20) | 4 (16) | 8 (32) |
| 3 | 6 (24) | 3 (12) | 0 (0) | 0 (0) | 0 (0) | 1 (4) | 1 (4) | 1 (4) | 0 (0) | 4 (16) |

**TABLE S2|** The max DSA MFI of ABMR patients in the diagnostic study.

| MFI intensity | Low MFI (1000-5000) | Moderate MFI (5000-10000) | High MFI (>10000) |
| --- | --- | --- | --- |
| Number of recipients | 9 | 6 | 10 |

**TABLE S3|** The lesion score of ABMR patients in the nested case-control study.

| c | g,  n (%) | ptc  n (%) | t  n (%) | i  n (%) | v  n (%) | cg  n (%) | ct  n (%) | ci  n (%) | cv  n (%) | C4d  n (%) |
| --- | --- | --- | --- | --- | --- | --- | --- | --- | --- | --- |
| 0 | 0 (0) | 0 (0) | 2 (25) | 6 (75) | 8 (100) | 8 (100) | 1 (13) | 1 (13) | 2 (25) | 3 (37) |
| 1 | 7 (87) | 7 (87) | 6 (75) | 2 (25) | 0 (0) | 0 (0) | 5 (62) | 5 (62) | 5 (62) | 4 (50) |
| 2 | 1 (13) | 1 (13) | 0 (0) | 0 (0) | 0 (0) | 0 (0) | 2 (25) | 2 (25) | 1 (13) | 1 (13) |
| 3 | 0 (0) | 0 (0) | 0 (0) | 0 (0) | 0 (0) | 0 (0) | 0 (0) | 0 (0) | 0 (0) | 0 (0) |

**TABLE S4|** The max DSA MFI of ABMR patients in the nested case-control study.

| MFI intensity | Low MFI (1000-5000) | Moderate MFI (5000-10000) | High MFI (>10000) |
| --- | --- | --- | --- |
| Number of recipients | 9 | 3 | 3 |
